# A Case of Musical Hallucinations in the Elderly

**DOI:** 10.1192/bjo.2025.10782

**Published:** 2025-06-20

**Authors:** Anastasia Tew

**Affiliations:** HPFT, Hertfordshire, United Kingdom

## Abstract

**Aims:** New onset psychosis in the elderly is rare with lifetime prevalence rates of 0.3–1%. Schizophrenia occurs in 0.1–0.5% of the elderly, however more common causes of new onset psychosis include dementia, delirium, drug-induced psychosis, and primary psychiatric disorders, most commonly depression. More than 50% of patients with Alzheimer’s disease experience psychotic symptoms and 70% develop delusions, in particular, persecutory type. Visual hallucinations are the most common type of hallucination occurring in 80% of patients with Lewy Body dementia. Second person auditory hallucinations occur in schizophrenia-like psychosis, however musical hallucinations where one perceives music without an external source are rare.

**Methods:** An 84-year-old female patient with no psychiatric background reported a 2-month history of musical hallucinations, causing distress and poor sleep. Patient denied hearing voices and other modalities of hallucinations. The musical hallucinations were not lateralised to either ear or side of space but experienced as external. Delirium was ruled out and patient’s medications were screened for anticholinergics and sedative-hypnotics. Patient had good cognition and a CT head excluded organic causes. The patient appeared distracted by the musical hallucinations, however, was not agitated and her mood was euthymic with congruent affect. No evidence of mood disorders ruled out psychotic depression and other affective disorders with psychotic symptoms. No thought disorders or delusions were present. Antipsychotics quetiapine and amisulpride proved no benefit for the patient.

**Results:** The patient’s presentation showed no clear reversible pathology and was unresponsive to antipsychotics. Musical hallucinations was given as a diagnosis of exclusion.

A musical hallucination is a type of auditory hallucination involving the perception of music without an external source. Patients are usually female, over 60 and subdivided into those with hyperacusis, psychiatric disorders, focal brain lesions, epilepsy and intoxication. Our patient was female and over 60 with presbycusis.

Musical hallucinations are proposed to be due to abnormal autonomous activity in the auditory brain systems responsible for normal music imagery. Sensory auditory deprivation or acquired peripheral deafness leads to impoverished auditory input and spontaneous activity occurs within the network for the perception and imagery of music.

**Conclusion:** This case report describes a rare phenomenon of musical hallucinations in an elderly patient. It identifies important diagnoses to exclude including delirium, dementia and other organic causes. The patient was female, over 60 yrs and diagnosed with presbycusis, characteristic of patients with musical hallucinations. The patient showed no improvement with antipsychotics and so treatment consisted of psychoeducation.

